# Exposure to *Treponema pallidum* among Female Sex Workers: A Retrospective Study Conducted in the State of Pará, Brazilian Amazon

**DOI:** 10.3390/pathogens13070559

**Published:** 2024-07-03

**Authors:** Thaís Mayara da Silva Carvalho, Paula do Socorro de Oliveira da Costa Laurindo, Diogo Oliveira de Araújo, Ricardo Roberto de Souza Fonseca, Rogério Valois Laurentino, Jacqueline Cortinhas Monteiro, Rosimar Neris Mantins Feitosa, Fernando Augusto Miranda da Costa, Leonardo Miranda dos Santos, Aldemir Branco Oliveira-Filho, Luiz Fernando Almeida Machado

**Affiliations:** 1 Biology of Infectious and Parasitic Agents Post-Graduate Program, Federal University of Pará, Belém 66075-110, PA, Brazil; thaissmcv@gmail.com (T.M.d.S.C.); paula.biomedica@gmail.com (P.d.S.d.O.d.C.L.); diaraujo84@gmail.com (D.O.d.A.); 2 Virology Laboratory, Institute of Biological Sciences, Federal University of Pará, Belém 66075-110, PA, Brazil; ricardofonseca285@gmail.com (R.R.d.S.F.); valois@ufpa.br (R.V.L.); jacqueline@ufpa.br (J.C.M.); rosimar@ufpa.br (R.N.M.F.); 3 Bacteriology Laboratory, Institute of Biological Sciences, Federal University of Pará, Belém 66075-110, PA, Brazil; fernandoamc@ufpa.br (F.A.M.d.C.); leonn_bio20@yahoo.com.br (L.M.d.S.); 4 Study and Research Group on Vulnerable Populations, Institute for Coastal Studies, Federal University of Pará, Bragança 68600-000, PA, Brazil; olivfilho@ufpa.br

**Keywords:** *Treponema pallidum*, sexually transmitted infections, female sex workers, epidemiology, public health

## Abstract

Background: Syphilis remains a significant global public health issue, and female sex workers (FSWs) are highly vulnerable to the etiological agent of this disease. This study aimed to describe the prevalence of exposure to *Treponema pallidum*, as well as the vulnerability factors among FSWs in the state of Pará, Brazilian Amazon. Methods: A cross-sectional, retrospective study involving 360 FSWs from five cities in Pará was conducted from 2005 to 2007. Blood samples were collected for treponemal and non-treponemal testing, and epidemiological information was obtained through interviews. Results: The exposure rate to *T. pallidum* was 37.7% (136/360), and the majority of FSWs had serological results indicating past exposure (21.1%). Among the FSWs exposed to *T. pallidum*, most of them were single, aged 23 to 42 years old, had less than 8 years of schooling, and had a family income of between 1 and 3 minimum wages. They reported using condoms during sexual intercourse and had no history of sexually transmitted infection (STI). Furthermore, many of the FSWs exposed to *T. pallidum* reported having more than 20 sexual partners per month, and had partners from other Brazilian states, but not from other countries. An age over 42 years and a reduced level of education were factors associated with exposure to *T. pallidum*. Finally, a high rate of exposure to *T. pallidum* among FSWs in the Brazilian state of Pará (from 2005 to 2007) was detected. In later years, epidemiological studies conducted with FSWs recorded that this rate remained high. Measures to control, treat, and prevent syphilis among FSWs were necessary between 2005 and 2007, and they are still imperative today. Actions related to educational programs and STI control, treatment, and prevention measures contained in Brazilian policies aimed at women’s health have not changed the vulnerability scenario of FSWs regarding their exposure to *T. pallidum*, even after 16 years, and must be reviewed and adapted to the conditions of the Brazilian Amazon.

## 1. Introduction

Syphilis is a systemic sexually transmitted infection (STI) caused by the bacterium *Treponema pallidum* subspecies *pallidum* (*T. pallidum*). Despite being curable, syphilis still represents a significant public health problem worldwide, given the high prevalences recorded in poor and developing countries and the increasing incidence detected in the population of men who have sex with men in developed countries [1,2]. *T. pallidum* can be transmitted from one person to another during sex (anal, vaginal, or oral) without a condom, through direct contact with primary and secondary syphilis lesions, or blood transfusion. Another form of transmission of this pathogen occurs from an infected mother to their child during pregnancy or childbirth [2].

The prevalence and incidence of syphilis has increased among men and women in different parts of the world [3,4,5]. A high prevalence of syphilis has been recorded in men and women in Latin America and the Caribbean (around 1.3%), and the highest incidence rate of this STI was observed in women and men in the Americas (5.3 cases per 1000 in men and women, which equates to over 3 million incident cases). In Brazil, there has been a significant upward trend in the prevalence of syphilis in its various forms from 2007 to 2017, representing a major public health risk in various municipalities located in the five Brazilian regions, showing that the challenge of reducing or even eliminating syphilis is still very difficult [5].

In this context, female sex workers (FSWs), as well as transgender women and transvestites, are considered key populations for acquiring syphilis and other STIs due to their high social and economic vulnerability, considering the high levels of stigma, violence, and criminalization. In Brazil, there are few epidemiological studies on syphilis in FSWs. In 2016, a multicenter study conducted with FSWs working in 12 Brazilian cities indicated a syphilis prevalence of 8.5% [6]. In three cities in the south of Brazil, the syphilis prevalence recorded among FSWs was 19.7% [7]. On the other hand, a high prevalence of syphilis has been detected in studies conducted with FSWs in the north of Brazil—36.1% in the cities of Belém, Macapá, and Rio Branco [8]; 14.1% in the municipalities of Augusto Corrêa, Barcarena, and Belém [9]; 41.1% in municipalities and riverside communities in the Marajó Archipelago [10]; and 36.9% in municipalities crossed by major highways in Pará [11]. Unprotected sex, multiple sexual partners, early initiation into sex work, illicit drug use, limited knowledge about STIs, and reduced monthly income have been associated with syphilis in this key population [12,13]. Therefore, investments in healthcare, health promotion, prevention, and social assistance for these women should be a priority for managers in Brazilian municipalities and states. Treating a disease like syphilis that affects different populations, such as pregnant women and FSWs, requires effective and integrated measures from healthcare services at all levels of complexity.

Since 1984, the Ministry of Health has developed women’s health assistance programs in Brazil, which have included educational, preventive, diagnostic, treatment, and recovery actions, encompassing women’s care in gynecological clinics, prenatal, childbirth, and postpartum care, menopause, family planning, STIs, and cervical and breast cancer, as well as other needs identified from the population profile of women [14,15]. However, there are still several gaps and challenges to be overcome regarding women’s health, such as adolescent women’s health, occupational health, mental health, infectious diseases, and the inclusion of gender perspective [15].

These challenges are even more difficult to overcome in the northern region of Brazil, a rural and socioeconomically underdeveloped area with limited transportation infrastructure and inadequate healthcare services, where poverty, malnutrition, domestic and urban violence, sex work, and the use and trafficking of illicit drugs are commonly observed and recorded at high levels [16]. Thus, this study describes the prevalence and factors associated with the exposure to *T. pallidum* in FSWs working in municipalities in the state of Pará, in the Brazilian Amazon.

## 2. Materials and Methods

### 2.1. Study Characteristics and Ethical Aspects

**Figure 1:**
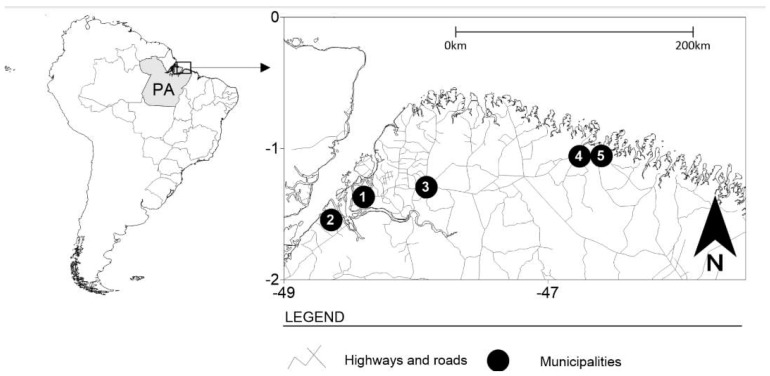
The geographic location of the municipalities where personal information and biological samples were collected from FSWs in northern Brazil. Points 1 to 5 are municipalities in the state of Pará (PA)—Belém (1), Barcarena (2), Castanhal (3), Bragança (4), and Augusto Corrêa (5).

The present study was cross-sectional, retrospective, descriptive, and analytical, involving FSWs from five municipalities in the state of Pará, northern Brazil—Belém (the capital), Bragança, Augusto Corrêa, Barcarena, and Castanhal (Figure 1). These municipalities are areas of an intense flow of people and the circulation of products with many historical, cultural, and tourist attractions that stand out in the context of the Amazon region, and, at the same time, they also have records of sexual trade in the state of Pará [10,11,12,13,16,17]. This study was approved by the Human Research Ethics Committee of the HEMOPA Foundation, under number 12/2005.

### 2.2. Study Design and Sampling

This study involved FSWs from January 2005 to August 2007. A non-probabilistic (convenience) sample was utilized to collect data from participants, with enrollment taking place four times a week at their workplaces (such as bars, streets, strip clubs, etc.), determined through sample size analysis. Women who identified as cisgender and engaged in sexual activities in exchange for money in the selected locations for at least six months were included in the study. Those who self-identified as transgender, women under the influence of alcohol or drugs during data collection, and those unable to respond to the epidemiological questionnaire were excluded from the study. Initially, a survey of prostitution venues in the evaluated municipalities was conducted, followed by approaching FSWs who played a leadership role in enhanced participant recruitment. They were invited to participate in the study after the presentation of the study.

All FSWs were informed about the study objectives and were invited to participate. Those who agreed to take part in the research signed an Informed Consent Form and provided epidemiological information. The refusal rate to participate in the study was 10.3%. The determination of the sample size was based on the estimated prevalence of syphilis in women in Belém, Pará (33.0%) at the time of the study. The sample error (ε) assumed in the calculation was 5%, and a test power of 80% was established, resulting in a minimum sample size of 320 FSWs.

### 2.3. Laboratory Tests and Interpretations

From each FSW, a sample of peripheral blood (5 mL) was collected using a vacuum collection system into tubes containing EDTA as an anticoagulant. Plasma was separated using centrifugation (9500 rpm for 15 min) and was stored at −20 °C until use at the Virology Laboratory of the Institute of Biological Sciences at the Federal University of Pará. FSW samples were examined for the detection of anti-Treponema pallidum antibodies (immunoglobulin M and immunoglobulin G) using the enzyme-linked immunosorbent assay (ELISA; Eti-Treponema Plus—DiaSorin, Cypress, CA, USA) according to the manufacturer’s instructions. The use of the ELISA to detect treponemal antibodies is considered to be a treponemal test, as it involves the detection of specific anti-treponemal antibodies in a sample [18]. FSWs with non-reactive results, as determined using the ELISA, were classified as not being exposed to *T. pallidum*, and no procedure or test was performed. All FSWs with reactive results according to the ELISA were classified as being exposed to *T. pallidum* (i.e., they have been exposed in the past or recently to *T. pallidum*) and had biological samples collected to perform additional tests. Reactive samples according to the ELISA were tested using the rapid plasma reagin (RPR-Brás-Laborclin, Paraná, Brazil). RPR is considered a non-treponemal test, as it detects antibodies that are not specific for *T. pallidum*, but that are found in patients with syphilis. All samples were tested pure (1:1) and in titrations (≥1:2) to eliminate the possibility of the prozone phenomenon in the execution of this laboratory test. The positivity of the sample in the treponemal test (ELISA) was considered as demonstrating an exposure to *T. pallidum* (outcome). All FSW samples were examined 15 to 30 days after collection.

### 2.4. Data Collection and Statistical Analysis

All FSWs completed an interviewer-administered questionnaire consisting of questions about sociodemographic, behavioral, drug use, and clinical/health outcome variables. These data were fed into an Excel database and were converted to STATA format for all procedures and statistical analyses. Significant associations between the outcome (with or without exposure to *T. pallidum*) and epidemiological information (sociodemographic, behavioral, drug use, and health/clinical variables) were assessed using the Chi-square test. The latter used a 0.05 significance level for the type I error. The data were analyzed using STATA 17 (StataCorp^®^, College Station, TX, USA).

## 3. Results

The study included 360 FSWs, with a mean age of 36.2 years (ranging from 15 to 71 years), with the majority (61.1%; 220/360) falling within the age range of 23 to 42 years. Regarding marital status, most participants were single (71.4%; 257/360), had less than 8 years of education (72.5%; 261/360), and had a family income of between 1 and 3 minimum wages (55.6%; 200/360).

The majority of study participants reported using condoms in all sexual encounters in the past 12 months (55.8%; 201/360), stated no history of STIs (78.1%; 281/360), but admitted to using illicit drugs (51.7%; 186/360) and having more than 20 sexual partners per month (54.4%; 196/360). Furthermore, 58.0% (209/360) reported having had partners from other states in Brazil, and 43.9% (158/360) had previously had partners from other countries (Table 1).

Overall, the prevalence of exposure to *T. pallidum* was 37.7% among FSWs. The rates of past exposure (21.1%) were higher than those detected for recent exposure (16.7%) to *T. pallidum* (Table 2). Among the FSWs exposed to *T. pallidum*, the majority of them were single, aged 23 to 42 years old, had less than 8 years of schooling, and had a family income of between 1 and 3 minimum wages. They reported using condoms during sexual intercourse, and had no history of STI. Furthermore, many of the FSWs exposed to *T. pallidum* reported having more than 20 sexual partners per month, and had partners from other Brazilian states, but not from other countries. Only two factors associated with exposure to *T. pallidum* were detected here—being aged over 42 years and having a reduced level of education (less than eight years of study). Other sociodemographic, economic, behavioral, and health factors were not associated with exposure to *T. pallidum* (Table 1).

The rates of past infections were higher than those detected for recent infections with *T. pallidum*. The overall prevalence of recent infections was 16.7%. In this study, nine (2.5%) FSWs showed positive results for RPR (with low titers) and negative results for ELISA. These cases were considered cross-reactions and were interpreted as false-positive results (i.e., FSWs are also susceptible to infection with *T. pallidum*). The majority of FSWs (59.7%) participating in this study had negative serology for anti-*T. pallidum* in both serological tests (Table 2).

Furthermore, significant associations were observed between syphilis infection and several demographic and behavioral factors among the FSWs. These factors included age groups, with a higher proportion of infections being found among those aged 23–42 years (65.5%, *p* < 0.001), and marital status, with a higher proportion of infections being detected among single individuals (76.5%, *p* = 0.144). Additionally, a significant association was found between syphilis infection and education level, with a higher proportion of infections being found among individuals with less than 8 years of study (79.4%, *p* = 0.007). These findings highlight the importance of considering socio-demographic factors in the prevention and control of syphilis among FSWs.

## 4. Discussion

The present study documented a high prevalence of exposure to *T. pallidum* (37.7%) among FSWs working in five municipalities in the Brazilian state of Pará, including the state capital, from 2005 to 2007, indicating the historical vulnerability of these women to *T. pallidum*. High rates of syphilis were recorded among FSWs after 2007. In the Marajó archipelago, state of Pará (2015–2017), a high prevalence of syphilis (41.1%) was recorded among FSWs in 25 locations (7 municipalities and 18 riverside communities), the majority of whom were young (18 to 30 years old), which differs from the findings in this study [10]. A high prevalence of syphilis (36.9%) was also recorded among FSWs who worked on the road system in the state of Pará from 2015 to 2016 [11]. This demonstrates that the history of syphilis and the profile of FSWs working in this Brazilian state in the Brazilian Amazon are very worrying; an increase in the number of syphilis cases and younger FSWs with syphilis has been reported.

The prevalence of exposure to *T. pallidum* among FSWs in the present study (including both new and old cases) was much higher than that found in other regions of Brazil, such as sex workers in Curitiba from 2010 to 2019 (1.0%) [19]; in 12 Brazilian cities in 2016 (8.5%) [6]; and in Tubarão, Laguna, and Imbituba, Southern Brazil (19.7%) in the year 2009 [7,20]. Our results demonstrate that exposure to *T. pallidum* in the state of Pará is much higher than that found in other populations of sex workers and non-sex workers, such as among residents of peri-urban islands in Belém, Pará, from 2020 to 2021 (5.9%) [21]; among recyclable waste collectors in Central Brazil from 2014 to 2016 (7.91%) [22]; and among manual sugarcane cutters (2.5%) in the Midwest and northeast regions of Brazil in 2016 [23].

Among Latin American countries, the prevalence of syphilis among FSWs in the present study was also higher than that found among FSWs in Lima, Peru (3.4%) [24]; in Argentina between 2006 and 2009 (22.4%) [25]; and in Colombia between 2001 and 2002 (10.3%) [26]. It is worth noting that in Argentina between 2000 and 2002, the prevalence of syphilis in FSWs was 45.7% [27]. However, in 1999, the prevalence was 2.4% in Venezuela [28]. Compared to other countries, the prevalence of syphilis found in Pará is much higher than that observed among FSWs in Ethiopia from 2019 to 2021 (6.2% and 11.3%) [29,30]; in China from 2013 to 2021 (1.8%, 1.73%, and 4.41%) [31,32,33]; in Kurdistan, west of Iran, from 2019 to 2020 (1.0%) [34]; in Togo in 2017 (0.8%) [35]; in Cameroon from 2015 to 2016 (8.3%) [36]; in the Middle East and North Africa in 2018 (12.7%) [37]; in Sudan and Uganda from 2015 to 2017 (7.3% and 9.2%) [38,39]; in Moscow, Russia, from 2017 to 2018 (13.9%) [40]; and in the Sino–Vietnam border area from 2016 to 2021 (8.8%) [41]. However, the prevalence of syphilis in the present study was like that found in Bangladesh at 38.2% [42] and was relatively higher than that observed in FSWs in Malawi in 2019 (9.7%) [43].

Additionally, a low level of education and a high age of FSWs were associated with exposure to *T. pallidum* in this study. The predictive role of lower educational levels, low monthly income, and longer involvement in the sex trade (representing older age) as factors associated with syphilis point to the role of adverse socioeconomic determinants and, specifically, social marginalization. This has been recorded in epidemiological studies on syphilis and STIs conducted in Brazil and other countries in South America [9,11,13,28,44,45]. Overall, the risk factors associated with syphilis here are clear indicators of the health vulnerability of these women in the Brazilian Amazon and, consequently, reflect the failure or absence of actions related to educational programs and measures of control, treatment, and prevention of STIs that are contained in the National Policy for Comprehensive Women’s Health Care, guiding women’s health care actions from 2004 to 2007 in Brazil [14,15]. Unfortunately, studies conducted on syphilis and other STIs among FSWs in the Brazilian Amazon after 2007 have reinforced the historical invisibility of FSWs about the National Policy for Comprehensive Women’s Health Care (PNAISM) and the National Plan for Women’s Policies (PNPM) in Brazil [8,9,10,11,44,45]. An example of this is the absence of any indicator (so far) to record, monitor, and track the living and health conditions of FSWs in Brazil.

This study has several limitations that should be considered. The sample size could have been larger. However, due to the significant social stigma surrounding FSWs worldwide, recruiting participants for studies aimed at promoting physical and mental health is very challenging, and the refusal rate is very high. Many women report feeling ashamed, and their families are unaware of their actual work, making it difficult to even deliver test results for treatment, as they do not provide their data correctly. Another relevant limitation is the origin of the data. As the interview data are self-reported, some information, such as drug use or sex-related risks behaviors, may contain response or recall bias. Additionally, the study used a convenience sample and may not establish a causal relationship. Although the information on exposure to *T. pallidum* and the profile of FSWs in this study pertains to the years 2005 to 2007, and the regional scenario of this pathogen and syphilis is probably different today, this study can serve as a basis for evaluating the dynamics of this STI over time, being an important tool for evaluating the effectiveness of public health policies and tackling the spread of *T. pallidum*.

## 5. Conclusions

A high rate of exposure to *T. pallidum* among FSWs in the Brazilian state of Pará (from 2005 to 2007) was detected. In later years, epidemiological studies conducted with FSWs recorded that this rate remained high. Measures to control, treat, and prevent syphilis among FSWs were necessary between 2005 and 2007 and, unfortunately, are imperative today. Actions related to educational programs and STI control, treatment, and prevention measures contained in Brazilian policies aimed at women’s health have not changed the vulnerability scenario of FSWs regarding exposure to *T. pallidum*, even after 16 years. The actions to be carried out in this group of vulnerable women must consider different aspects, from the level of education and economic capacity to risk behaviors for acquiring STI and the trauma caused by the violence experienced.

## Figures and Tables

**Table 1:** Demographic, socioeconomic, behavioral, and health characteristics of FSWs in the Brazilian state of Pará, from January 2005 to August 2007, related to exposure to *T. pallidum*.

Characteristics	Total (n = 360)	No Exposure (n = 224)	With Exposure * (n = 136)	*p*-Value
	N	n (%)	n (%)	
Age groups (years)				<0.01
15–22	112	86 (76.8)	26 (23.2)	
23–42	220	131 (59.5)	89 (40.5)	
>42	28	7 (25.0)	21 (75.0)	
Marital status				0.09
Single	257	153 (59.5)	104 (40.5)	
Married	56	35 (62.5)	21 (37.5)	
Widow/divorced	47	36 (76.6)	11 (23.4)	
Education level				0.02
<8 years of study	261	153 (58.6)	108 (41.4)	
>8 years of study	99	71 (71.7)	28 (28.3)	
Family income (in minimum wage)				0.07
Less than 1	131	89 (67.9)	42 (32.1)	
From 1 to 3	200	114 (57.0)	86 (43.0)	
More than 3	29	21 (72.4)	8 (27.6)	
Condom use				0.27
Yes	201	120 (59.7)	81 (40.3)	
No	159	104 (65.4)	55 (34.6)	
STI ^#^ history				0.06
Yes	79	42 (53.2)	37 (46.8)	
No	281	182 (64.8)	99 (35.2)	
Use of illicit drugs				0.13
Yes	186	126 (67.7)	60 (32.3)	
No	174	98 (56.3)	76 (43.7)	
Number of partners (monthly)				0.45
<20	164	106 (64.6)	58 (35.4)	
≥20	196	118 (60.2)	78 (39.8)	
Partners from other states of Brazil				0.19
Yes	209	124 (59.3)	85 (40.7)	
No	85	60 (70.6)	25 (29.4)	
Do not know	66	40 (60.6)	26 (39.4)	
Partners from other countries				0.83
Yes	158	97 (61.4)	61 (38.6)	
No	202	127 (62.9)	75 (37.1)	

* Treponemal test positive (ELISA) = exposure to *T. pallidum.* ^#^ STI = sexually transmitted infection.

**Table 2:** Prevalence of exposure to *T. pallidum* (etiological agent of syphilis) among female sex workers in the state of Pará, Brazilian Amazon.

Prevalence (Serological Markers)	% (Positive/Total)	95% Confidence Intervals
Exposure to *Treponema pallidum* (IgG * + IgM **)	37.7 (136/360)	32.8–42.8
Past exposure (IgG *)	21.1 (76/360)	16.9–25.3
Recent exposure (IgM **)	16.7 (60/360)	12.8–20.5

* IgG = Immunoglobulin G; ** IgM = Immunoglobulin M.

## Data Availability

Data are contained within the article.
